# Human renal adipose tissue induces the invasion and progression of renal cell carcinoma

**DOI:** 10.18632/oncotarget.21666

**Published:** 2017-10-09

**Authors:** Fiorella Campo-Verde-Arbocco, José D. López-Laur, Leonardo R. Romeo, Noelia Giorlando, Flavia A. Bruna, David E. Contador, Gastón López-Fontana, Flavia E. Santiano, Corina V. Sasso, Leila E. Zyla, Constanza M. López-Fontana, Juan C. Calvo, Rubén W. Carón, Virginia Pistone Creydt

**Affiliations:** ^1^ Instituto de Medicina y Biología Experimental de Cuyo (IMBECU), Centro Científico y Tecnológico Mendoza, Consejo Nacional de Investigaciones Científicas y Técnicas (CONICET), Mendoza, Argentina; ^2^ Clinica Andina de Urología, Mendoza, Argentina; ^3^ Departamento de Urología y Transplante Renal, Hospital Español, Mendoza, Argentina; ^4^ Instituto de Biología y Medicina Experimental (IBYME), Buenos Aires, Argentina; ^5^ Departamento de Química Biológica, Facultad de Ciencias Exactas y Naturales, Universidad de Buenos Aires, Buenos Aires, Argentina; ^6^ Centro de Medicina Regenerativa, Facultad de Medicina, Clinica Alemana, Universidad del Desarrollo, Santiago, Chile; ^7^ Universidad Nacional de Cuyo, Facultad de Ciencias Médicas, Mendoza, Argentina

**Keywords:** human adipose tissue, renal epithelial cells, cancer, epithelial-stromal interactions, migration

## Abstract

We evaluated the effects of conditioned media (CMs) of human adipose tissue from renal cell carcinoma located near the tumor (hRATnT) or farther away from the tumor (hRATfT), on proliferation, adhesion and migration of tumor (786-O and ACHN) and non-tumor (HK-2) human renal epithelial cell lines. Human adipose tissues were obtained from patients with renal cell carcinoma (RCC) and CMs from hRATnT and hRATfT incubation. Proliferation, adhesion and migration were quantified in 786-O, ACHN and HK-2 cell lines incubated with hRATnT-, hRATfT- or control-CMs. We evaluated versican, adiponectin and leptin expression in CMs from hRATnT and hRATfT. We evaluated AdipoR1/2, ObR, pERK, pAkt y pPI3K expression on cell lines incubated with CMs. No differences in proliferation of cell lines was found after 24 h of treatment with CMs. All cell lines showed a significant decrease in cell adhesion and increase in cell migration after incubation with hRATnT-CMs *vs.* hRATfT- or control-CMs. hRATnT-CMs showed increased levels of versican and leptin, compared to hRATfT-CMs. AdipoR2 in 786-O and ACHN cells decreased significantly after incubation with hRATfT- and hRATnT-CMs *vs.* control-CMs. We observed a decrease in the expression of pAkt in HK-2, 786-O and ACHN incubated with hRATnT-CMs. This result could partially explain the observed changes in migration and cell adhesion. We conclude that hRATnT released factors, such as leptin and versican, could enhance the invasive potential of renal epithelial cell lines and could modulate the progression of the disease.

## INTRODUCTION

An essential information exchange is established between epithelial tissue and fibroblastic/adipose stroma for both normal morphogenesis and functionality, as well as for cancer development. Therefore a deep understanding of epithelial-stromal interactions is fundamental in order to understand and control these processes. Perirenal adipose tissue is one of the most abundant cell types that surround renal epithelial cells.

Adipose tissue acts as an endocrine organ, secreting soluble factors and contributing to the composition of the extracellular matrix (ECM) [1, 2]. Specifically, it can secrete numerous inflammatory intermediaries such as ILs (IL-1β, IL-6 y IL-10), chemokines (IL-8 o CXCL8, CCL2 o MCP-1 y CXCL10), growth factors (HGF, NGF, VEGF and TNFα) and adipokines (such as leptin and adiponectin). Peripheral and visceral adipose tissue secrete different sets of growth factors and cytokines [3], several of which have been seen to influence significantly the progression of cancer and other diseases [2, 4-8].

Renal cancer or renal cell carcinoma (RCC), is considered the fifth most frequent cancer type worldwide, and is related to a high mortality rate in both men and women [9]. In Argentina an average of 4100 new cases are reported every year, with a higher incidence in men (SIVER/INC based on data from Globocan 2012. Argentina, 2016). The most frequent type is clear cell renal carcinoma [10]. Genetic alterations, excessive and prolonged alcohol consumption, inadequate working conditions, smoking and even obesity, have been positively related to this cancer type [8]. Recent studies have shown that adipocytes and tumor cells interact significantly within the invasive front [11, 12]: tumor cells modify adipocytes, and as a consequence, adipocytes increase the metastasic ability of tumor cells [6].

Leptin is a hormone that regulates adipocyte size and the development of some tumor types. Its involvement in tumor development would appear to be related to its antiapoptotic, mitogenic and proangiogenic effects. An over-expression of leptin receptors (ObR) has been recently found in some cancer types [13]. Furthermore, leptin promotes the invasiveness of murine renal cancer cells via extracellular signal-regulated kinases and Rho guanosine triphosphatase dependent pathways [14]. Thus, leptin signaling might have a key role in renal cell carcinoma invasion.

In addition, adiponectin regulates glucose and fatty acid metabolism, modifying physiological processes such as eating behavior and hematopoyesis. ObR is involved in protumorogenic pathways, while adiponectin receptors (AdipoR1 and AdipoR2), possess proangiogenic and antiapoptotic effects [2]. In RCC patients, adiponectin levels are reduced and correlate inversely with the size of the tumor [8]. Even more, increased metastatic potential is associated with a reduction in AdipoR2 levels [15]. Both leptin and adiponectin are produced within adipose tissue and seem to have opposite effects on the regulation of cancer [16].

One of the candidate factors is versican, a chondroitin sulphate proteoglycan that is essential in epithelial cell-ECM interaction and intracellular signaling. In some tumors, increased levels of versican have been found, which suggests that it might contribute to cancer progression [17, 18]. Recently, Zi X *et al.* [19] demonstrated that secreted factors from perineoplasm perinephric adipose tissue (PAT) may play a role in facilitating metastasis or perirenal fat invasion of clear-cell renal carcinoma (ccRCC) by mobilizing ccRCC cells away from primary tumor sites.

Our group has recently focused on the study of human adipose tissue samples from mammary and prostate, as well as kidney. The analysis of human tissue samples is of great importance, since animal adipocytes share several common properties with human fat cells, but also exhibit substantial differences, such as in factors affecting insulin resistance. Our group has demonstrated that conditioned media (CMs) from periprostatic tissue of tumoral prostates influence tumoral behavior even during initial stages of the disease [20]. Recently, we have seen that proliferation, adhesion and migration of breast cancer epithelial cell lines are regulated by CMs from human breast cancer adipose tissue explants (hATT) [7].

In the present study, we evaluated the effects of CMs of human adipose tissue explants from renal cell carcinoma near the tumor (hRATnT) or farther away from the tumor (hRATfT), on proliferation, adhesion and migration on tumor (786-O and ACHN) and non-tumor (HK-2) human renal epithelial cell lines. Additionally, we aim to characterize factors that are modified: 1) in hRATnT and hRATfT; and 2) in 786-O, ACHN and HK-2 cell lines when incubated with CMs from hRATnT and hRATfT.

## RESULTS

### Proliferation of 786-O, ACHN (tumor) and HK-2 (non-tumor) cells is not modified by hRATnT- or hRATfT-CMs

Protein quantification (total amount) was performed in the conditioned media: hRATnT-CMs: 1.33 ± 0.12 μg/μl (n=10), and hRATfT-CMs: 1.02 ± 0.11 μg/μl (n=6).

**Figure 1:**
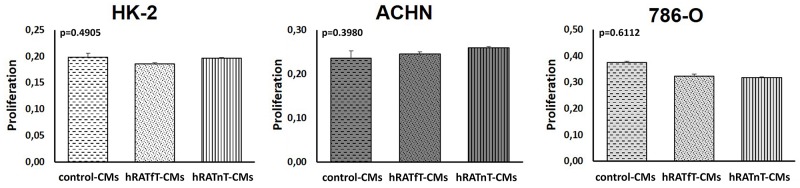
Effect of CMs from hRATnT and hRATfT on proliferation of HK-2, ACHN and 786-O cell lines HK-2, ACHN and 786-O cell lines were incubated with hRATnT- (n=10), hRATfT- (n=6) or control-CMs for 24 h. Proliferation was measured by MTT assays. Data are shown as the mean ± SEM (n = 4-5 experiments by triplicate).

In order to identify proliferation and dead cells both MTT technique and cell counting with Tripan blue respectively were assessed, finding in both cases consistent results. After incubating 24 h with hRATnT-, hRATfT- or control-CMs, proliferation was not modified in any of the cell lines studied (Figure 1).

The same assays were performed incubating 48 and 72 h with CMs. No differences in proliferation were found (data not shown).

### Adhesion of 786-O, ACHN (tumor) and HK-2 (non-tumor) cells is decreased by hRATnT-CMs

**Figure 2:**
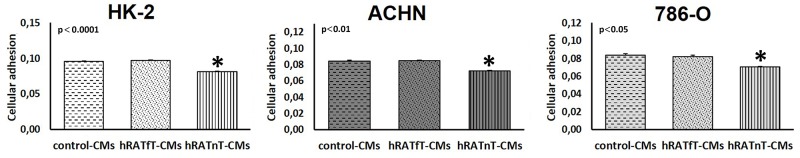
Effect of CMs from hRATnT and hRATfT on HK-2, ACHN and 786-O cell lines attachment HK-2, ACHN and 786-O cell lines were plated at a density of 5x10^4^ cells/well in wells preincubated ON with hRATnT- (n=8-10), hRATfT- (n=3-6) or control-CMs and adherent cells were quantified by MTT. Data are shown as the mean ± SEM (n = 3 experiments by triplicate). ^*^p < 0.05 hRATnT-CMs *vs.* hRATfT-CMs and control-CMs.

786-O, ACHN and HK-2 cells were seeded in plates previously exposed to different CMs. hRATnT-CMs significantly reduced the adhesion of cells compared to hRATfT-CMs (Figure 2, p<0.05). On the other hand hRATfT-CMs did not affect 786-O, ACHN or HK-2 cell adhesion *vs.* control-CMs (Figure 2).

### Migration of 786-O, ACHN (tumor) and HK-2 (non-tumor) cells increased after incubation with hRATnT-CM

**Figure 3:**
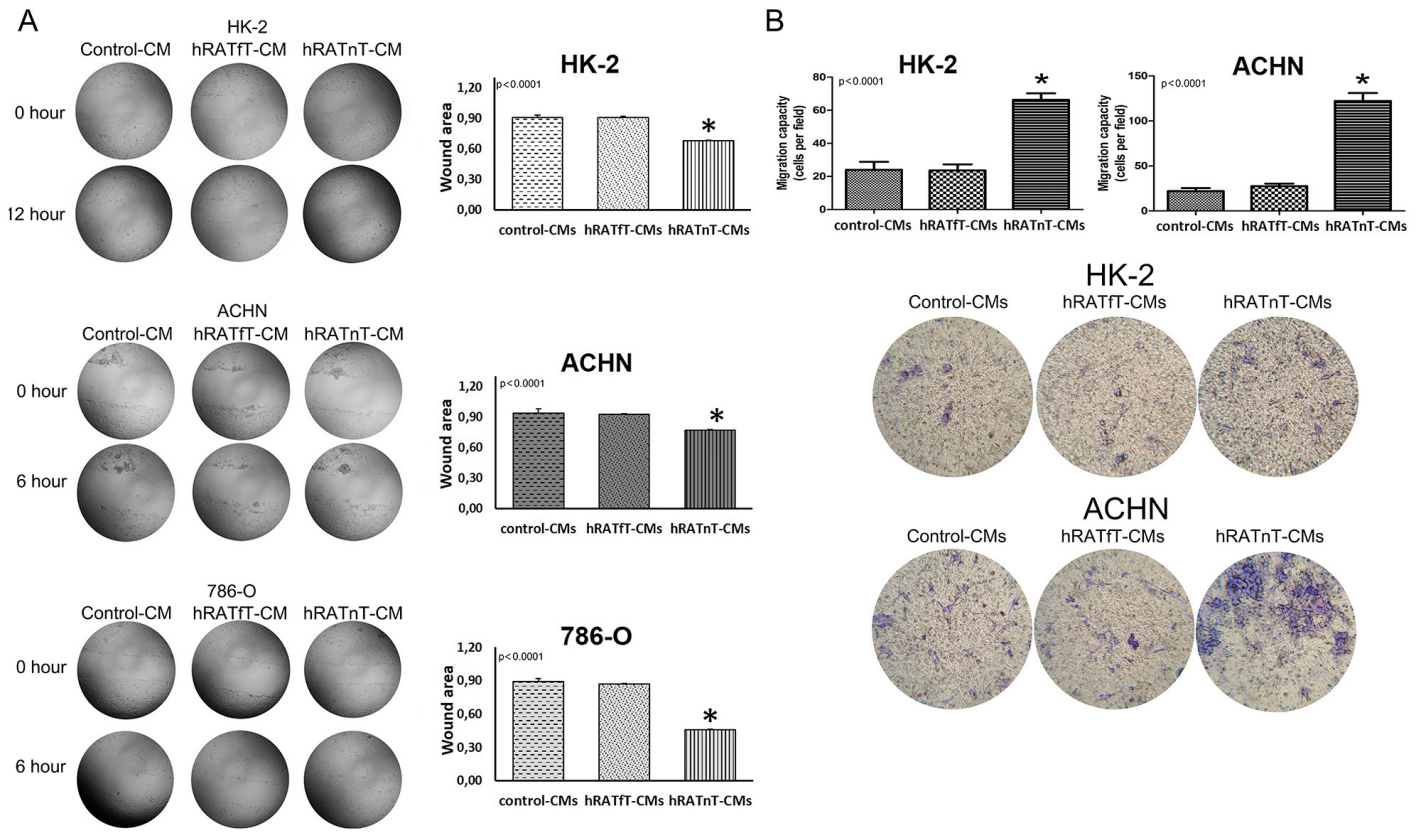
Effect of CMs from hRATnT and hRATfT on migration of HK-2, ACHN and 786-O cell lines Wound healing assay **(A):** HK-2, ACHN and 786-O cell lines were grown hRATnT- (n=8-10), hRATfT- (n=3-6) or control-CMs by additional 24 h. After that, cells were wounded, washed twice with PBS and hRATnT- (n=8), hRATfT- (n=4) or control-CMs were added. Images were captured at the wound instant (0 h), after 6 h, and after 12h. Representative light microscopic images (4x) are shown. HK-2, ACHN or ACHN were incubated with hRATnT-, hRATfT- or control-CMs. The histogram shows the ratio of 12 h/0 h (HK-2) or 6 h/0 h (ACHN and 786-O) cutting area and is plotted as mean ± SEM (n = 3 experiments by duplicate). ^*^p < 0.0001 hRATnT-CMs *vs.* hRATfT-CMs and control-CMs. Transmigration assay **(B)**: HK-2 and ACHN cells were incubated with hRATnT- (n=8-10), hRATfT- (n=3-6) or control-CMs allowed to migrate across the porous membrane for 24 h. The membranes were viewed under 20X magnification and migrated cells were counted in 5 randomly chosen fields per membrane. Data are shown as the mean ± SEM (n = 3 experiments by duplicate). ^*^p < 0.0001 hRATnT-CMs *vs.* hRATfT-CMs and control-CMs.

hRATnT-CMs increased significantly migration of 786-O and ACHN after incubating for 6 h (p<0.0001), as well as migration of HK-2 (non-tumor cell) after incubating for 12 h (p<0.0001), *vs.* the effect of hRATfT-CMs and control-CMs (Figure 3A). Transwells migration assays results showed a similar pattern: transmigration of HK-2 and ACHN cells increased significantly when incubated with hRATnT-CMs *vs.* the effect of hRATfT-CMs (p<0.0001) and control-CMs (p<0.0001) (Figure 3B).

### Compared to hRATfT, hRATnT showed an increase in versican mRNA expression, while adiponectin and leptin mRNA expression was not modified

**Figure 4:**
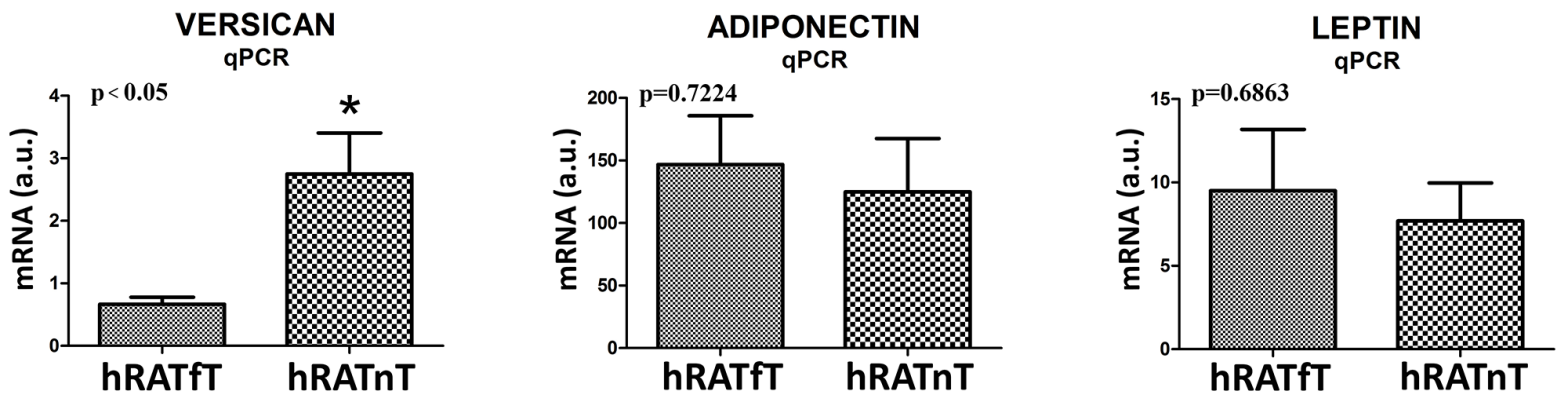
Versican, adiponectin and leptin gene expression from hRATnT and hRATfT The mRNA profiles of versican, adiponectin and leptin from different adipose tissue were analyzed by RT-PCR and normalized by their relative ratio to GAPDH. Data are mean ± SEM. GAPDH, glyceraldehyde-3-phosphate dehydrogenase. ^*^p<0.05.

We measured genetic expression (mRNA levels) of versican, adiponectin and leptin in adipose tissue far away (hRATfT) and near (hRATnT) tumor cells. Results showed an increased level of versican mRNA in hRATnT compared to hRATfT (Figure 4, p<0.05). Although no significant differences were found in adiponectin and leptin mRNA, a tendency to increase was seen in the hRATnT-CMs (Figure 4).

### Compared to hRATfT-CMs, hRATnT-CMs showed an increase in versican and leptin expression, while adiponectin expression was not modified

**Figure 5:**
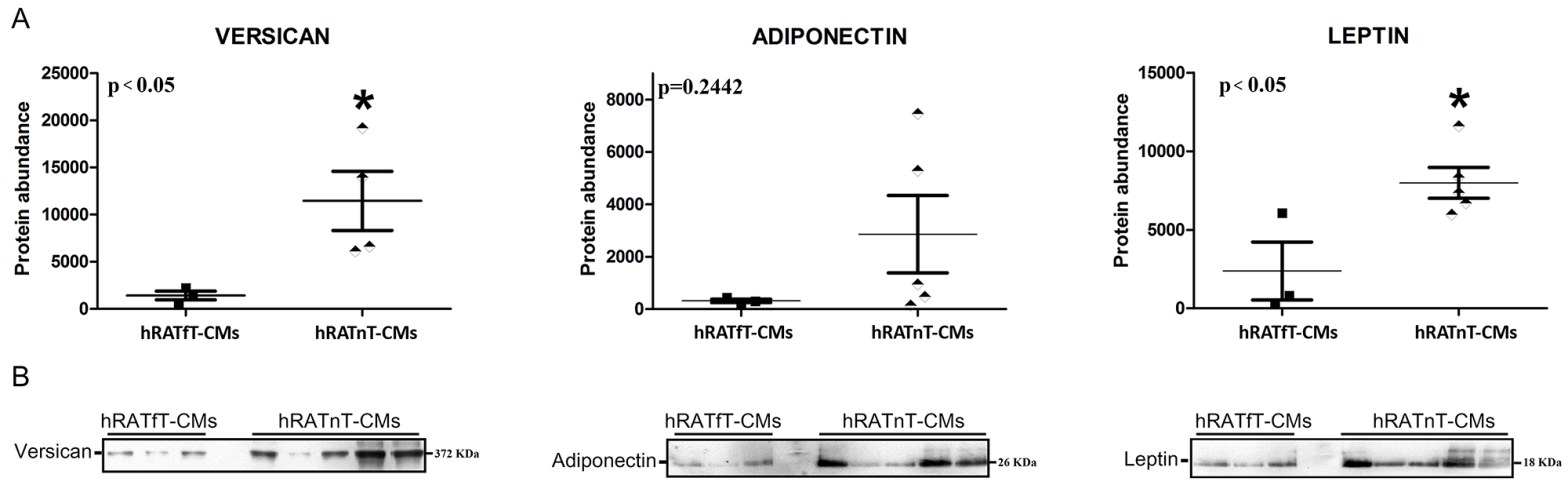
Versican, adiponectin and leptin in hRATnT- and hRATfT-CMs **(B)** Versican, adiponectin and leptin expression was evaluated by Western blot. Images were analyzed by densitometry. **(A)** Horizontal bars represent the geometric mean of each data set. Vertical bars indicate SEM. ^*^p < 0.05 hRATnT-CMs *vs*. hRATfT-CMs.

Results showed that leptin and versican expression increased significantly in hRATnT-CMs *vs.* hRATfT-CMs (Figure 5, p<0.05). In the case of adiponectin, expression showed a non-significant increase in hRATnT-CMs (Figure 5, p=0.24).

### An increase in versican and leptin expression was found in hRATnT adipocytes compared to hRATfT adipocytes

**Figure 6:**
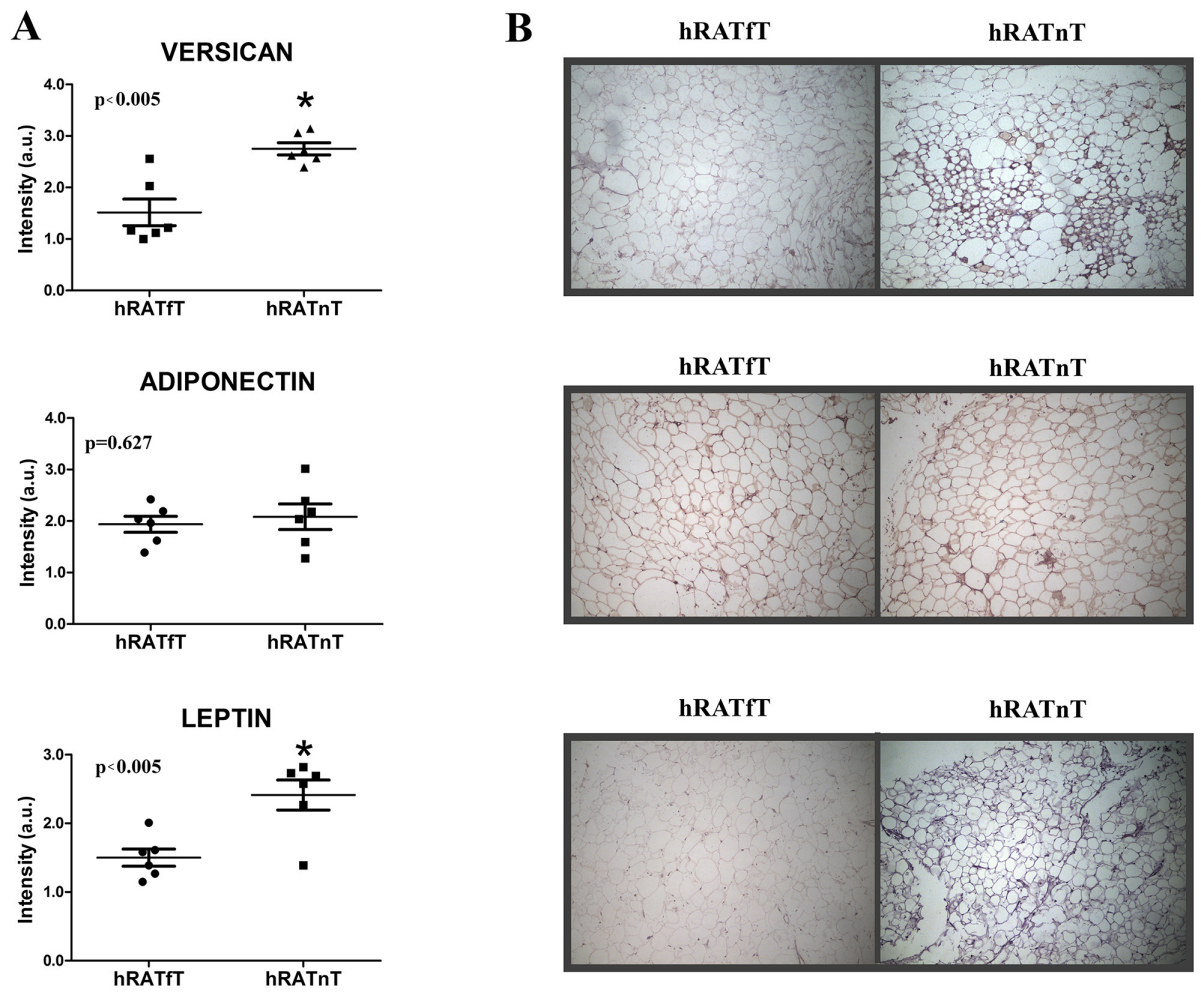
Versican, adiponectin and leptin expression in the different adipose tissues Versican, adiponectin, and leptin expression was evaluated by immunohistochemistry in serial cuts of hRATnT and hRATfT. DAB staining quantification in the three tissue types was performed with Image J software (NIH). Histograms **(A)** show mean ± SEM of six independent experiments. (a.u.: arbitrary units). ^*^p < 0.005 hRATnT *vs.* hRATfT. Representative photographs **(B)** of hRATnT- and hRATfT-staining. Magnification: ×100.

We performed an immunohistochemistry assay on adipose tissue far away (hRATfT) and near (hRATnT) tumor cells, in order to measure versican, adiponectin and leptin levels and localization in both tissue samples. We found increased levels of versican and leptin expression in hRATnT adipocytes (Figure 6, p<0.005). However, when we evaluated the levels of adiponectin, we did not find a significant difference between the tested samples (Figure 6). These results correlate with protein levels previously obtained by Western blot.

### In renal cancer cells hRATfT- and hRATnT-CMs decrease AdipoR2 expression, but do not change AdipoR1 and ObR expression

**Figure 7:**
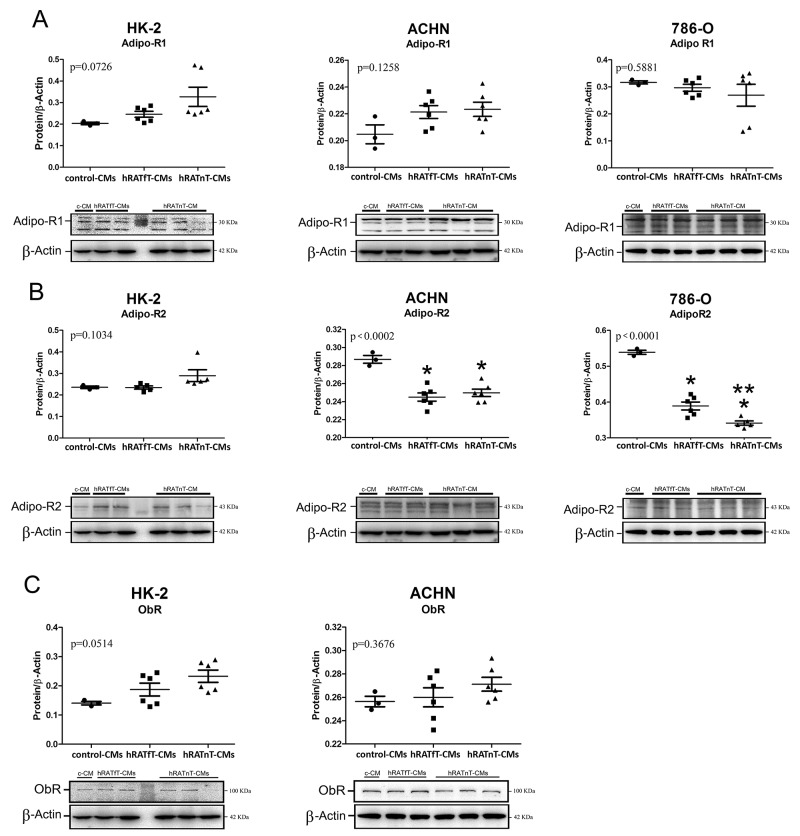
Effect of CMs from hRATnT and hRATfT on: AdipoR1 **(A)**; AdipoR2 **(B)** and ObR **(C)** expression was evaluated in HK-2, ACHN and 786-O cell lines. HK-2, ACHN and 786-O cells were grown on 6 well plates, incubated for 24 h with the different CMs and then lysed. Expression of the different proteins was measured by Western blot. β-actin was used as internal control. Images were analyzed by densitometry Horizontal bars represent the geometric mean of each data set. Vertical bars indicate SEM. ^*^p < 0.001 tumor cells incubated with hRATnT- or hRATfT-CMs vs. control-CMs; ^**^p < 0.05 tumor cells incubated with hRATnT-CMs vs. hRATfT-CMs.

A decrease of AdipoR2 expression was found in 786-O and ACHN (tumor cells) incubated with hRATfT- and hRATnT-CMs *vs.* control-CMs (Figure 7B, p<0.001). In addition, AdipoR2 expression in 786-O cells was significantly lower when these cells were incubated with hRATnT-CMs compared to hRATfT-CMs. Nevertheless, no significant changes were observed in AdipoR1 or ObR expression in any of the three cell lines incubated with the different CMs (Figure 7A and 7C).

### hRATnT-CMs decrease pAkt expression but do not change pERK expression in renal cancer cells; decrease pPI3K expression in tumor cells; and increase pPI3K expression in non-tumor cells

**Figure 8:**
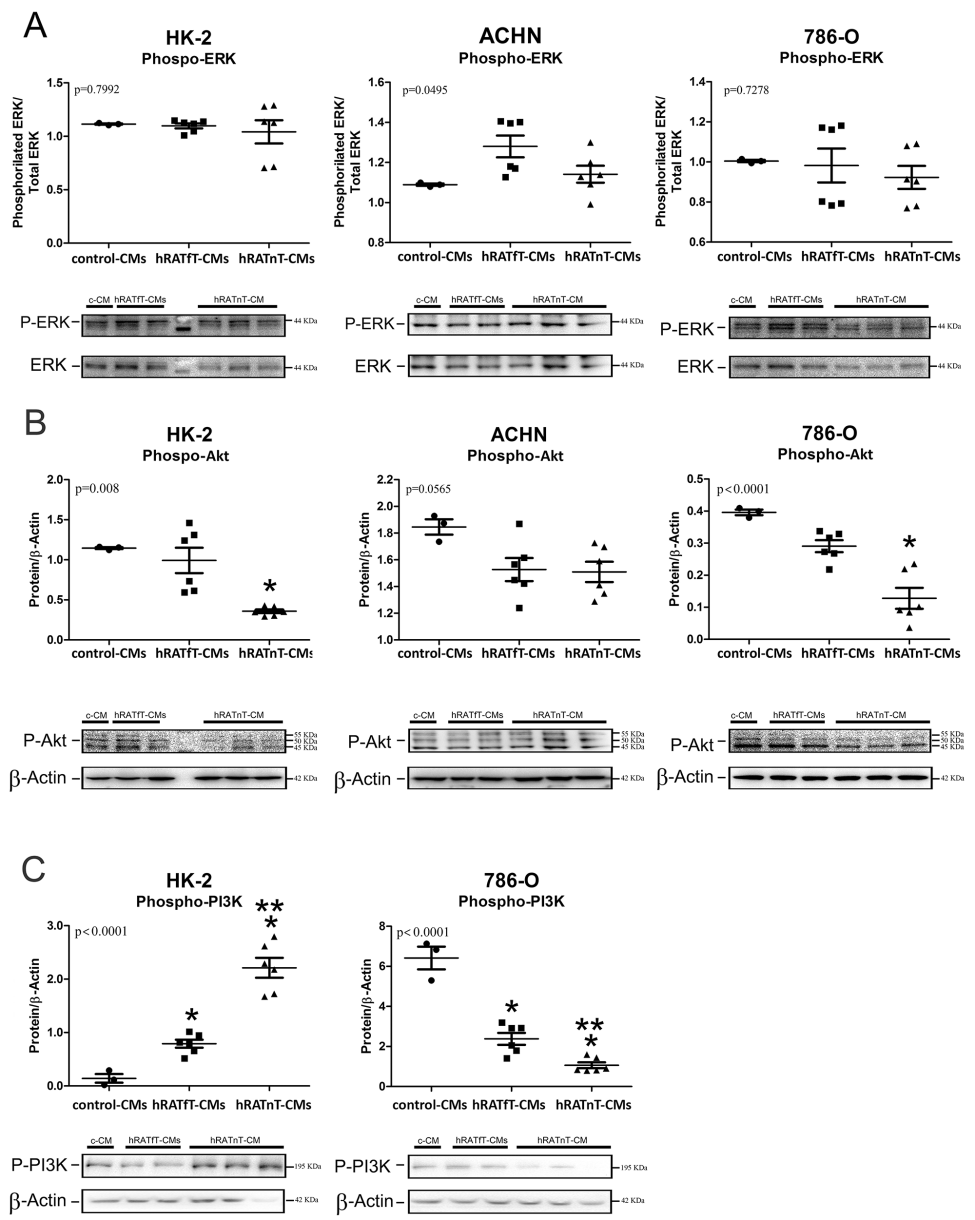
Effect of CMs from hRATnT and hRATfT on pERK **(A)**, pAkt **(B)** and pPI3K **(C)** expression was evaluated in HK-2, ACHN and 786-O cell lines. HK-2, ACHN and 786-O cells were grown on 6 well plates, incubated for 24 h with the different CMs and then lysed. Expression of the different proteins was measured by Western blot. β-actin was used as internal control. Images were analyzed by densitometry Horizontal bars represent the geometric mean of each data set. Vertical bars indicate SEM. ^*^p < 0.05 cells incubated with hRATnT- or hRATfT-CMs vs. control-CMs; ^**^p < 0.05 cells incubated with hRATnT-CMs vs. hRATfT-CMs.

In order to begin elucidating the intracellular mechanisms involved in the observed biological effects (such as changes in cell adhesion and migration), pERK, pAkt and pPI3K expression was evaluated. No changes in p-ERK were observed after incubation with the different CMs. We found a significant decrease of pAkt expression in all three cell lines incubated with hRATnT-CMs *vs.* hRATnT- and control-CMs (Figure 8B). This result was statistically significant in the case of HK-2 and 786-O cells (p<0.05), and marginally significant in the case of ACHN cells. Furthermore, pPI3K expression in 786-O cells decreased significantly when these cells were incubated with hRATnT-and hRATfT-CMs *vs.* control-CMs (Figure 8C, p<0.001). An opposite result was found in HK-2 cells: pPI3K expression was significantly higher in HK-2 cells incubated with hRATnT-and hRATfT-CMs compared to control-CMs (Figure 8C, p<0.001).

## DISCUSSION

The involvement of adipocytes during tumor development and metastatic capacity, has only recently begun to be investigated [21]. In humans, adipose tissue can be found surrounding several organs such as the kidneys, and in subcutaneous connective tissue, being overall the most common type of tissue [8]. Therefore, in the present work we aimed to evaluate the possible role of adipose tissue microenvironment in the control of the physiology of renal epithelial cells. Thus, we evaluated the effects of conditioned media of human adipose tissue explants from renal cell carcinoma near the tumor (hRATnT) or farther away from the tumor (hRATfT), on cell proliferation, adhesion, migration of 786-O, ACHN and HK-2 cell lines. Furthermore, we characterized factors that are altered in tumor and non-tumor human renal epithelial cell lines, after incubation with hRATnT- and hRATfT-CMs. These factors could be involved in the biological effects that were observed. Expression of AdipoR1, AdipoR2, and ObR was evaluated. Furthermore, expression of several intracellular proteins involved in various cascades activating transcription of genes, such as pERK, pAkt and pPI3K, was evaluated. We also measured expression of versican, leptin and adiponectin in hRATnT-CMs *vs* their expression in hRATfT-CMs by different methods.

Results indicate that human adipose tissue CMs from renal cell carcinoma near the tumor differentially regulate the adhesion and migration of renal tumor and non-tumor epithelial cell lines, *vs.* CMs from renal cell carcinoma farther away from the tumor. Thus, results show that adipose tissue near the tumor is able to regulate tumor and non-tumor epithelial cell adhesion to the stroma, and participates in the metastatic ability of renal epithelial cells. The obtained results agree with those published by Zi X et al. [19], who observed increased motility of RCC incubated with CMs from perineoplasm perinephric adipose tissue. In the present work we employed not only human renal epithelial cell lines, but also a non-tumoral cell line (HK-2). Results showed that hRATnT-CMs increases not only the metastasic potential of tumor cells, but also migration capacity of non-tumor cells.

In the present work we analyzed the possible contribution of various soluble factors separately of possible direct cell contact effects. Versican is a constituent of the ECM; and it has been adduced that it might be implied in the regulation of mechanisms such as angiogenesis, cell migration and proliferation. Different studies show a strong relation between the expression of versican and a more aggressive and metastatic behavior in different cancer models [18, 23]. We found increased levels of versican mRNA and protein expression in CMs from hRATnT *vs.* CMs of hRATfT. Accordingly, Horiguchi *et al.* [14] demonstrated that the cytokine leptin derived from adipocyte was able to stimulate Renca renal cancer cell invasiveness, activating ERKs and rho GTPase. This result agrees with the finding in the present study of increases in leptin expression in CMs of hRATnT *vs.* CMs of hRATfT. Thus, Figures 4, 5 and 6 show, by different methods, that adipose tissue behaves differentially in relation to its proximity to tumoral tissue. Furthermore, this might point out that versican and leptin might increase the metastatic ability of renal cancer cells. Leptin and versican are secreted by white adipose, located in whole body, and can in an endocrine, paracrine and autocrine manner, promote cancer proliferation and survival. Nevertheless, in in the present work we aimed to analyze whether renal adipose tissue behaved differentially in relation to its proximity to renal tumor cells. Our results show that indeed adipose tissue taken near the tumor secretes (among other factors), increased levels of leptin, which has been shown to have tumorigenic properties. Thus, in addition to possible effects of leptin secretion by white adipose from other parts of the body, what we see in the present work is that adipose tissue near the tumor shows increased levels of leptin secretion (*vs.* adipose tissue taken farther away from the tumor), thus enhancing local tumorigenic effects. Additionally, our results show a decrease in AdipoR2 expression in 786-O and ACHN (tumor cells) incubated with hRATfT- and hRATnT-CMs *vs.* control-CMs. AdipoR2 expression in 786-O cells was significantly lower when cells were incubated with hRATnT-CMs compared to hRATfT-CMs. Different studies have shown reduced adiponectin levels in RCC patients, and an inverse correlation between adiponectin level and tumor size [8]. In the present study, we found increased adiponectin expression in hRATnT-CMs *vs.* hRATfT-CMs, although the difference did not achieve statistical significance. Thus, tumor cells seem to down-regulate their response to adiponectin by decreasing the expression of AdipoR2 in the cell membrane. Pinthus *et al.* [15] demonstrated that reduced AdipoR2 levels are associated with increased metastatic potential. We found differences of AdipoR2 expression in 786-O and ACHN that might be associated to differences in the origin of both cell lines: while 786-O is derived from a primary clear cell adenocarcinoma (primary tumor); ACHN is a line derived from pleural effusion, i.e. a metastatic site. This difference could also explain why ObR expression was found in ACHN cells (although levels were not modified with the different CMs), but not in 786-O cells. In addition, we evaluated changes in intracellular signal pathways (phospho-ERK, phospho-Akt and Phospho-PI3K) of normal and tumor cell lines after incubation with the different conditioned media. Results showed differential expression of these intracellular signal pathway proteins in all cell lines after incubation with the CMs from adipose tissue taken far away (hRATfT) and near (hRATnT) tumor cells. We observed a decrease in the expression of pAkt in HK-2, 786-O and ACHN incubated with hRATnT-CMs. This decrease of the active (phosphorilated) form of Akt could at least partially explain the observed changes in migration and cell adhesion. Finallly, we found a significant increase of pPI3K in HK-2 cells incubated with hRATfT- and hRATnT-CMs compared to control. This increase was higher with hRATnT-CMs vs. hRATfT-CMs. In 786-O cells we observed the opposite effect: a significant decrease of pPI3K when incubated with hRATfT- and hRATnT-CMs compared to control.

In conclusion, results seem to show that tumor microenvironment dynamically interacts with the epithelium. Characterizing the factors that are involved in this interaction might allow to develope new procedures to prevent and treat renal cancer.

## MATERIALS AND METHODS

### Reagents

Reagents were from Sigma Chemical Co (St. Louis, MO, USA), tissue culture flasks, dishes, and multi-well plates were from Falcon Orange Scientific (Graignette Business Park, Belgium), and culture media from both tissue and cell lines and supplements were from Gibco BRL (Carlsbad, CA, USA).

### Sample collection and handling

Human adipose tissue explants from renal cell carcinoma near the tumor (hRATnT; n=10) or farther away from the tumor (renal pole opposite tumor; hRATfT; n=6) were obtained from patients with renal cell carcinoma (RCC), that underwent radical or partial nephrectomy; who had not received previous chemotherapy or radiotherapy treatment. The median of body mass index (BMI) of the patients was 22.8 kg/m2. and both adipose tissue samples (taken far away and near the tumor) were from the same patient. (BMI) (kg/m2) was calculated as weight (kg) divided by height (m) squared.

Samples were transported in PBS with gentamicin (50 μg/ml) and processed immediately. On average, 2 h elapsed from the acquisition of the surgical sample until it was processed under a sterile laminar flow hood. The project was approved by the ethics committee of the Facultad de Ciencias Médicas de la Universidad Nacional de Cuyo, Argentina and all patients gave their informed consent to undergo tissue harvesting for this research.

### Preparation of conditioned media (CMs) from hRATnT and hRATfT

Adipose tissues were washed three times with cold PBS to remove red blood cells and debris, and weighed. hRATnT or hRATfT were plated in culture flask with M199 culture medium (Invitrogen™; 1 g tissue/10 ml M199) supplemented with gentamicin (50 μg/ml) and incubated for 1 h at 37°C in 5 % CO_2_. After that, the medium was removed and replaced with fresh medium and the tissues were incubated for 24 h. Subsequently, the supernatants were collected and the cells were removed by centrifugation (3 min at 400 x g). Then, the supernatants were aliquoted into 1-ml fractions and immediately stored at -80°C. The control-CMs were obtained from the collection of serum free M199 medium after 24 h of incubation in a culture flask at 37°C in 5 % CO_2_.

### Treatment with hRATnT- and hRATfT-CMs

In order to study proliferation, migration and expression of different proteins of tumor (786-O and ACHN) and non-tumor (HK-2) human renal epithelial cell lines, MCs collected were diluted 1:1 in DMEM-F12 (Invitrogen, UK) 2 % fetal bovine serum (FBS; 1 % FBS final concentration) and the cells were incubated with the diluted CMs. The experiments were performed with equal volumes of hRATnT- and hRATfT-CMs. The concentration of total protein in those volumes was quantified using Bradford reagent.

### Culture of tumor and non-tumor renal epithelial cell lines

Tumor (786-O and ACHN) and non-tumor (HK-2) human renal epithelial immortalized cell lines were used. 786-O ([7860] (ATCC® CRL1932™), ACHN (ATCC® CRL1611™) and HK-2 (ATCC® CRL2190™) were obtained from the American Type Culture Collection (ATCC, Rockville, MD). 786-O is a line derived from a primary clear cell adenocarcinoma (primary tumor); and ACHN is a line derived from pleural effusion (metastatic site). The three cell lines were cultured in DMEM-F12 medium with 10 % FBS and 2 μg/ml insulin; and were maintained at 37°C in 5 % CO_2_. The number of generations for HK-2 was 12-15; 17-21 for 786-O, and 10-13 for ACHN.

### Proliferation assay

Tumor (3x10^3^ 786-O or ACHN cells/well) and non-tumor (5x10^3^ HK-2 cells/well) human renal epithelial cell lines were incubated on 96-well plates with complete DMEM-F12 for 24 h. Then, the three cell lines were treated with hRATnT-, hRATfT- or control-CMs. After 24 or 48 h, MTT was added to each well at a final concentration of 1 mg/ml and incubated at 37°C for 2 h. The media/MTT solution was then removed without disturbing the attached cells, and accumulated formazan in cells dissolved in acidified (4% 1N HCl) isopropanol. The absorbance of each sample was determined at 570 nm. Each sample was repeated three times. Results are expressed as percentage of color intensity and normalized to cells grown in control-CMs. In addition, we evaluated cell proliferation by the cell count technique using Tripan blue in order to identify dead cells.

### Cell adhesion assay

Adhesion assays were performed following a protocol previously reported [22]. Briefly, 96-well plates were coated with 100 μl hRATnT-, hRATfT- or control-CMs at 37°C overnight in 5 % CO_2_. These CMs were set in three wells and each experiment was repeated three times. Plates were then blocked with 1 mg/ml bovine serum albumin at 37°C for 1 h. After washing with PBS, 786-O, ACHN and HK-2 cells (5x10^4^ cells/well) were suspended in serum-free DMEM-F12 medium, seeded and allowed to adhere to the CMs factors-coated wells at 37°C for 1 h in 5 % CO_2_. Non-adherent cells were aspirated and wells washed twice with PBS. Residual cells were evaluated by MTT assay. Cell adhesion to hRATnT-, hRATfT-CMs factors was expressed as percentage of control-CMs.

### Cell migration assay

The effect of hRATnT-, hRATfT- or control-CMs on the motility of tumor and non-tumor human renal epithelial cell lines was evaluated by wound-healing and by Transwells migration assays.

### Wound-healing assays

786-O, ACHN and HK-2 cells cells were grown on 96-well plates with complete DMEM-F12. Confluent cell monolayers were wounded with a pipette tip, washed twice with PBS and hRATnT-, hRATfT- or control-CMs were added. Images at time zero (0 hours) were captured to record the initial width of the wounds. The recovery of the wounded monolayers due to cell migration toward the denuded area was evaluated after 6, 12 and 24 h. The images were acquired by an inverted phase-contrast microscope (Olympus CKX-41) using a 4x objective. Quantification was performed using ImageJ (NIH, Bethesda, MD, USA) by a polygon selection mode and determining the percentage of the wounded area at 6 or 12 h respect to control (0 h).

### Transwells migration assays

HK-2 and ACHN cells (2 9 105 cells/0.2 ml) were placed into the top transwell with 8 lm pore membranes (NUNC cat.#140629). They were then incubated with hATN-, hATT- or control-CMs and allowed to transmigrate across the porous membrane for 20 h. At the end of the assay, inserts were removed and the cells were fixed in 4 % paraformaldehyde and then stained with a 0.1 % crystal-violet solution. Tumor and non-tumor cells on the upper membrane surface were removed with a paper towel. The air-dried membranes were viewed under 209 magnification and migrated cells were counted in 5 randomly chosen fields per membrane.

### mRNA expression analysis

Total RNA was isolated from adipose tissue using Trizol reagent (Invitrogen, Carlsbad, CA) and quantified by absorbance at 260 nm. One microgram total RNA was reverse transcribed with 200U M-MLV reverse transcriptase (Promega, Madison, WI) and 300 pmol oligo-dT. Real-time PCR was performed in 20 mL final volume containing 50 ng cDNA, PCR LightCycler-DNA Master SYBRGreen reaction mix (Roche, Indianapolis, IN), 4 mM MgCl_2_, and 0.5mM each specific primer using a LightCycler thermocycler (Roche, Indianapolis, IN). Controls without reverse transcription were included to ensure that amplification was from mRNA and not from genomic DNA. Amplicons were characterized according to their melting temperature, determined by LightCycler thermocycler, and after their size had been evaluated after being electrophoresed on agarose gel. Each target gene’s mRNA level was standardized against the GAPDH mRNA level for each sample. The Ct method was used for mRNA quantification, expressed as arbitrary units (a.u.).

### Preparation of cell lysates from renal epithelial cells after incubation with hRATnT-, hRATfT- or control-CMs

786-O, ACHN and HK-2 cells were seeded in six-well plates in DMEM-F12 complete medium. When cells reached 75-80 % confluence, the medium was aspirated and cells were washed twice with PBS. Then, cells were incubated at 37°C for 24 h either with hRATnT-, hRATfT- or control-CMs (50% CM, 50% DMEM-F12 2% FSB). Cells were lysed with Ripa buffer, pelleted by centrifugation at 4°C and stored at -80°C.

### Western blot analysis

#### Protein expression in epithelial cell lines

In order to evaluate protein expression levels, Western blots were performed. AdipoR1, AdipoR2, ObR, ERK, pERK, pAkt, and pPI3K were measured after incubation of the epithelial cell lines with the different CMs obtained. In order to lyse cells, Ripa buffer was used (Tris 10mM pH 7,5; NaCl 150mM; sodium vanadate 2mM; sodium deoxycholate; SDS 0,1%; igepal 1%; protease inhibitors). Total protein in samples was quantified by Bradford method. Proteins were separated in a SDS-PAGE 12 or 18% gel, and electrotransferred to a nitrocellulose membrane (Amersham). The membrane was later blocked with bovine serum albumin (Sigma-Aldrich, 0055K) and then incubated with the different antibodies ON at 4°C. The membranes were later washed, and incubated with proper secondary antibodies conjugated to HRP. Antibody complexes were visualized by means of chemiluminescence (ECL; GE Helathcare). Bands were quantified by densitometry using FIJI Image processing package [24]. In the cell extracts, β-actin level in samples was used to determine that equal quantities of proteins were loaded in the gel.

### Protein expression in CMs

In addition, versican, adiponectin and leptin expression was measured in hRATnT- and hRATfT-CMs. Total protein in samples was quantified by Bradford method. Proteins were separated in a SDS-PAGE 12 % gel, and electrotransferred, afterwards we performed the same protocol described for cells. The assay for the hRATnT- and hRATfT-CMs was done by loading equal volumes of each CM (40μl).

### Immunohistochemistry

Serial cuts were performed on the same tissue samples used for H&E staining. Versican, adiponectin, and leptin expression were studied by means of immunohistochemistry. Briefly, hRATnT and hRATfT microtome slides were first deparaffinized, and then a heat-mediated antigen retrieval, endogenous peroxidase blocking and nonspecific tissue blocking were performed. Slides were then incubated with the different primary antibodies at 4°C, and after that with an anti-rabbit biotinylated secondary IgG antibody. Finally, slides were incubated with peroxidase-conjugated streptavidin. Peroxidase reaction was performed with chromogen 3,3′-diaminobenzidine (DAB) (DAKO LSAB + Kit, HRP). Hematoxylin counter stain was performed. Serial cuts incubated in the absence of primary antibody were used as negative controls. Images were taken with a Nikon Eclipse E200 Microscope fitted with a Micrometric SE Premium (Nikon Corp., Japan) digital still camera at 100x and 400x magnifications. DAB staining quantification in the three tissue types was performed in 8–10 fields of each preparation as mentioned above.

### Statistical methods

The statistical significance between different experimental conditions was evaluated by *t-*test or one-way ANOVA. Tukey´s post-hoc tests were performed within each individual treatment. The results are presented as mean ± SEM. Results were considered significant at p < 0.05.
